# Theorizing disaster communitas

**DOI:** 10.1007/s11186-021-09442-4

**Published:** 2021-03-16

**Authors:** Steve Matthewman, Shinya Uekusa

**Affiliations:** 1 Department of Sociology, University of Auckland, 10 Symonds Street, Level 9, Auckland, 1010 New Zealand; 2 School of Culture and Society, Aarhus University, Aarhus, Denmark

**Keywords:** Altruism, Carnival, Communitas, Community of fate, Disaster risk reduction (DRR), Disasters, Social capital

## Abstract

Disaster scholars have long complained that their field is theory light: they are much better at doing and saying than analyzing. The paucity of theory doubtless reflects an understandable focus on case studies and practical solutions. Yet this works against big picture thinking. Consequently, both our comprehension of social suffering and our ability to mitigate it are fragmented. Communitas is exemplary here. This refers to the improvisational acts of mutual help, collective feeling and utopian desires that emerge in the wake of disasters. It has been observed for as long as there has been a sociology of disasters. Within the field, there have been numerous efforts to name and describe it. Yet there has been far less enthusiasm to theorize it, which means that the disaster literature has not adequately explained the social conditions under which communitas arises (or fails to). In this article, we synthesize numerous case studies to do so. This takes us beyond simple statements of what communitas is and what it should be called, to considerations of the conditions under which it emerges, how it should be conceptualized, the factors that might prevent communitas, and how we might encourage it. While primarily a theoretical work, the identification of communitas’ facilitators and barriers have practical import for disaster risk reduction (DRR) policy as communitas has frequently proven to be a positive and potent force.

Disaster scholars regularly criticize their collective efforts for being theory light. In his Presidential Address to the International Research Committee on Disasters, Robert A. Stallings (2006) expressed fears that, as a consequence, disaster researchers were fringe-dwellers within academia, doomed to “institutionalized marginality”. Other leading figures within the field have taken disaster studies theoretical stagnation to task for being isolationist, piecemeal and silent on issues of political power (Alexander, 2013; Dombrowsky, 1995, p.242; Kreps & Drabek, 1996, p.136). For Kathleen Tierney (2010, p.660), the only theoretical “progress” of note has been a shift from functionalism to mid-range theory.

Two formerly promising theoretical drives, around vulnerability and resilience, also seem to have come to dead ends.^1^ Work on vulnerability was initially welcomed for identifying the root causes of disaster and locating them within society. In addition to foregrounding structural factors, it also extended the spatial and temporal horizons of disaster research, considering such things as the ongoing legacies of colonization (Oliver-Smith, 1999a). However, across time, scholars increasingly problematized vulnerability discourse, condemning it for homogenizing, disempowering and pathologizing communities. Consequently, “it is now in danger of becoming simply a term to describe a set of immutable conditions” (Oliver-Smith, Forthcoming).

Research on vulnerability was increasingly replaced by work on resilience. Resilience was first formulated as the antithesis of, and solution to, vulnerability. This seemed to have the advantage of restoring agency to disaster victims and of opening up thinking about the ways in which disasters impact different people, and how they in turn respond and recover. However, its problems have been legion. A nebulous notion that sprang from multiple sources (including ecology and engineering), it has been difficult to define, conceptualize and measure (Manyena, 2006). Moreover, whereas vulnerability work may have obscured agency, resilience scholarship often occludes structure. This particularly applies to neoliberalized policy domains where the focus is firmly upon individualized adjustment rather than systemic reform. Thus, resilience can operate as yet another discourse which transforms public issues into personal troubles (Uekusa, 2018), and as with the vulnerability discourse, it can end up victim-blaming. One indicator of the declining scholastic purchase of resilience is that Taylor & Francis ceased publishing its eponymous journal in 2019. Given all of the above, Enarson et al., 2007 p.139) assessment that most disaster studies have been strong in practice (doing) and description (saying), but lacking in appropriate theory (analyzing) still stands.^2^

In this article, we make a modest contribution to offsetting our sub-discipline’s theory problem by synthesizing numerous case studies to analyze communitas. This takes us beyond simple statements of what communitas is and what it should be called, to the contributions that theorizing it may yield, identifying the reasons for its emergence, analyzing the affinities (and divergences) with related social science terms and social situations, noting the mediating factors that explain differential disaster recovery (the role of capitals) or even prevent it from developing, and thinking about how we might promote communitas. While primarily a theoretical work, the identification of communitas’ facilitators and barriers have practical import for disaster risk reduction (DRR) policy. We begin by defining the term.

## Communitas: What is it?

Communitas is the coming together of people for other people to secure a world together. It refers to the improvisational social bonds and spontaneous mutual support that arises within communities when disaster strikes (Jencson, 2001). It has been observed by researchers for as long as there has been a sociology of disasters. Samuel Henry Prince (1920) is the sub-field’s progenitor. He studied community reactions to the explosion of the French munitions ship Mont Blanc in Halifax, Nova Scotia. His analysis of that event revealed “the role of catastrophe in stimulating community service, in presenting models of altruistic conduct, in translating energy into action, in defending law and order, and in bringing into play the great social virtues of generosity, sympathy and mutual aid” (Prince, 1920, p.58). External aid filled 1700 train cars and five steamers. City officials received over 1000 offers to adopt orphaned children. Within the community, Prince (1920, pp.56–57) noted how volunteer firefighters had put their own lives at risk to prevent a second explosion. Longshoremen had done likewise, sailing other munitions ships to safety. Cafes gave meals away without charge. Pharmacists did the same with their supplies. The Grocer’s Guild coordinated with the relief committee to deliver food for free. Such was the outpouring of goodwill that Halifax was dubbed the City of Comrades.

Numerous studies across the subsequent century have come to the same conclusion: “disasters bring out prosocial and innovative behaviors in communities” (Knowles, 2011, p.213). But because communitas has been “discovered” so frequently, it is known by numerous terms. Other extant phrases include “emergency togetherness” (Drury et al., 2009), “extraordinary community” (Solnit, 2009), “post-disaster solidarity” and “the brotherhood of pain” (Oliver-Smith, 1999b), “post-disaster utopia” (Wolfensburg, 1957), “pro-social behavior” (Rodríguez et al., 2006), “social utopia” (Fritz, 1961), and “therapeutic community” (Barton, 1969).

Following Victor Turner (1969, 1986, pp.101–102), our preference is to use communitas.^3^ This is a Latin term that captures the pure spirit of community, denoting togetherness, bonding, collective action, and a society of equals. It also gestures towards two other significant elements of the phenomenon under study: a sense of shared joy and a utopian impulse. For in the depths of disaster, we may also see the outline of a different world, a desire to remake the world better.

## Why should we theorize communitas?

We think that it is important to theorize communitas for at least six reasons. First, as noted, disaster scholars themselves have frequently raised concerns about the poverty of theory within their domain.

Second, it is important to challenge the enduring power of “disaster mythology” within public discourse (Fischer, 2002). This is the prevalent belief that society breaks down during disasters, that people panic, and that the social fabric unravels into a war of all against all. As noted in the section above, the sociology of disasters provisions us with a century’s worth of empirical evidence showing that disaster situations do not conform to media stereotypes or the fears of the powerful. Anomic states are rare, anarchic ones are rarer still. Instead, people display high levels of other-directedness (Dynes & Quarantelli, 1977).

Third, while people and communities do not typically exhibit high-level alarm, the same cannot be said of the media and other authorities, as detailed by the literature on “elite panic” (Clarke & Chess, 2008; Lindahl, 2013). Examples of “recreancy”, the failure of institutional players to perform their roles responsibly and to the expected standards of proficiency, also abound (Freudenburg, 1997, p.33). Moreover, in disaster situations, authorities frequently align in the service of elite interests at the expense of public and environmental wellbeing (Molotch, 1970), leading some to argue that the greatest harms wrought to the social fabric are the predatory actions of the most powerful rather than those at the bottom of the social order (Klein, 2007; Loewenstein, 2015).

Fourth, since interests diverge, this makes the efficacy of solely relying upon top-down “command and control” models of disaster recovery extremely questionable (Tierney, 2014, p.203). We see a role for community empowerment. Stated simply, victims should be party to their own recovery. Indeed, this is regarded as best practice DRR (Twigg, 2015, p.115). “However, informal actors are rarely incorporated into formal disaster and humanitarian planning” (Twigg & Mosel, 2017, p.443).^4^ Enhancing the conditions that create communitas and recognizing its powerful potential is one way to achieve it. As Richardson et al. (2014) put it:

The presence of communitas may have a significant impact on a community’s recovery process following a disaster … [so] recognizing communitas as a useful framework for understanding successful and efficient disaster recovery in small communities may aid emergency management officials in the creation of effective short-term and long-term recovery strategies (p.212).

Fifth, scholarship and policy on disaster recovery overwhelmingly focuses upon physical “lifeline” infrastructures, those hard technological systems that sustain life, like communication, energy and transportation systems, rather than social infrastructure. Yet research suggests that it is social rather than physical infrastructure that is the ultimate driver of resilience (Aldrich & Meyer, 2015; Klinenberg, 2018). As such, its development is increasingly being recognized as a vitally important tool for disaster management and risk reduction (UN Chronicle, 2016). We discuss this in first subsection of “Communitas denied” below.

Finally, it is hoped that the discussion of communitas can make contributions to theorizing more broadly. By including the overlooked element of soft infrastructure, this article offers a contribution to the current “turn to infrastructure” (Amin, 2014), taking place across the social sciences.^5^

## Why does communitas emerge?

As noted, communitas is one of the most commonly made observations when disaster strikes (Hearn, 1980, p.300; Matthewman, 2015, p.166). In this section, we discuss the reasons for its emergence.

A survey of the literature reveals five key reasons for the development of communitas. First, it arises because disasters are social phenomena. Threats and damage are public and widely experienced. This sense of suffering together bonds survivors (Connell, 2001), leading to an “emergent shared social identity” (Drury et al., 2019).

Second, collective action is further encouraged as current power structures are nowhere near as robust as is commonly thought. They often dissipate or disappoint (Drabek & McEntire, 2003). Realization that official assistance is seldom in the right place at the right time in the right numbers gives civil society a boost. Mutual aid from within this “society of equals” may be the only resource available (Haney, 2018, p.106; Wood et al., 2013, p.143). Indeed, it is usually first responders, fellow citizens, who do the heavy lifting when disaster strikes (Tierney, 2003).

It is important for us to signal that this grassroots organization can be an awesome force. After Hurricane Katrina, the “Cajun Army” of volunteers was credited with saving thousands of lives (Tierney, 2014, p.203), more than 200,000 citizens offered shelter to strangers, and tens of thousands helped out with the Gulf Coast clean-up (Solnit, 2009, p.2). Similarly, the evacuation of Manhattan following the attacks on the World Trade Center facilitated by people who had access to boats was “the largest water evacuation in our history” (Perrow, 2007, p.297). We are currently witnessing a “superbloom of altruistic engagement” (Solnit, 2020) the world over as citizens collectively respond to COVID-19. In March 2020, for example, the British government called for a quarter of a million volunteers to come forward to assist senior citizens, those in isolation, and frontline medical staff who required supplies delivered. Over three times that amount offered to assist (Solnit, 2020).^6^

Third, the pronounced sense of agency is seen as a vital sense-making activity which has emotional and practical benefits. Adjustment to the “new normal” enhances both individual and collective wellbeing, a coping strategy that helps facilitate resilience and wellbeing (Chamlee-Wright & Storr, 2010). This must typically be done in the moment. “People … rarely develop the social interpretive capabilities required to understand novel and challenging events prior to disaster events occurring. Consequently, they have to do so during disaster response and recovery phases” (Paton & Irons, 2016).

Fourth, another major driver for collective action comes from the realization that disasters (triggered by natural hazards) are often experienced as “uncontrollable”. No one can be blamed (Kaniasty & Norris, 2004) – or, if they can, those persons, groups and organizations reside beyond the community. Instead of internal scapegoating and community division, there is a sense of togetherness (Casagrande et al., 2015), a “democracy of common disaster” (Wood quoted in Kutak, 1938, p.72).

Finally, undergirding all of this is the fact that we are essentially social beings. We cannot exist alone. We are products of culture and collective labor. We share norms and relations and are remarkably altruistic.^7^ As such, we are disposed to assist others (Peek 2020).^8^ Disasters throw this aspect of our species into sharp relief. As Drabek and Quarantelli (1967) concluded, disasters “often bring out the best in individuals. Ability to endure suffering, desire to help others, and acts of courage and generosity come forth in times of crisis” (p. 12).

## Clarifying communitas I: Cognate concepts

In this section and the one to follow, we clarify our thinking about communitas by considering its relationship to other social science constructs and social practices. We commence by comparing and contrasting it with the cognate terms “community of fate” and “social capital”.

### The community of fate

Communitas has many commonalities with the Weberian conception of a “community of fate”. Peter Baehr (2005, pp.185–191) elucidates. He notes that there are seven defining elements to such communities. Their strong group identity is based on: recognition of a shared and imminent hazard that must be addressed, moral density – which is to say high levels of interconnectedness stemming from this common threat and their shared interest in responding to it, the duration of the problem (a brief isolated event is unlikely to create such a community), closure – an inability to flee the situation, sufficient material and organizational resources to deal with the challenges, convergence – a common language or other points of connection, and social ritual (practices that provide the affected community with an identity, and that delineate these times from normal ones and the lives of those unaffected).

In making these points, Baehr distinguishes the community of fate from altruism and communitas. “In my sense, the solidarity instantiated by a community of fate is based not on altruism but on a focused sense of inter-connectedness and membership, not on love, but on danger, not on sympathy, but on fear” (Baehr, 2005, pp.202–203). Further, “[i]t is not to be confused with the communitas that, according to Victor Turner, accompanies the ritual process: a modality of social relationship that blends homogeneity, equality, comradeship. What homogeneity there is concerns the common danger and uncertainty” (Baehr, 2005, p.203). While communitas and communities of fate have similarities – reaction to a serious hazard, shared fate, unity of purpose – there are also significant points of difference. Communitas entails the suspension of hierarchy, the manifestation of a utopian impulse and, because of this, a carnivalesque element (which we elaborate upon in the next section). Instead of fear-based solidarity – which is a common argument for solidarity more generally in contemporary society (on this see Bauman, 2006; Beck, 1992, p.49; Furedi, 2005; Massumi, 1993, p.10; Virilio, 2012, p.35; and Žižek, 2008, p.79) – there is something more akin to the Durkheimian notion of collective effervescence: the suspension of normal rules, extraordinary togetherness, an emergent social energy, coordination of thoughts and actions, and a shared sense of empowerment (Smith 2020, pp.49–50).^9^

Indeed, Hearn (1980) wrote how “socio-cultural breakdown initially generates a feeling of euphoria and a sense of hopeful solidarity embodied in an idealized vision of a better society [as] important attributes of the context of communitas” (p.301). Similarly, Solnit (2005) wrote of disasters “as wonderful cessations of everyday life, in which people turned to one another for assistance and enjoyed the transformed spaces and practices” (p.31). Disaster scholars have long-seen what must be one of the most counter-intuitive occurrences in the social world: *joy* in disasters (Solnit, 2005, p.7, p.306). This is also reflected in media reports: community responses to an earthquake described as “positive” (Sinani quoted in Povoledo, 2016), responses to a train crash described as “beautiful” (Blaine quoted in Daley, 2016), and the same word used to describe the US public’s COVID-19 response (Graff, 2020).

### Social capital

While there are also clear affinities between communitas and social capital, as with the community of fate, there are notable points of difference too. Communitas occurs in conditions of what Victor Turner (1969) calls “anti-structure”, which is to say moments of social breakdown, or in exceptional circumstances, beyond quotidian experience. Social capital speaks to a more structured state. And, unlike communitas, social capital has a long-recognized dark side. While disaster researchers are wont to see it as an unqualified good, early sociological writers on social capital noted that the networks through which it accrues are often based on exclusions. This perpetuates hierarchies and secures ongoing structural advantage (Bourdieu, 1986). The strategic and self-interested investment/return motivations that define social capital (Colclough & Sitaraman, 2005, p.479) are lacking in communitas, which, as we have established, is substantially other-directed. Communitas is more of a public good. Social capital can work against the public good in that it restricts some from access to resources (because only connected members are eligible). Indeed, social capital can even produce social bads as in organized crime. There are additional points of difference. Communitas speaks to a society of equals sharing a common experience. Social capital does not. Communitas is place-based while social capital networks may be geographically dispersed. Communitas is the spontaneous formation of new bonds rather than the strengthening of pre-existing ones. Under conditions of communitas, social bonds are strong, to the point of being intoxicating (Kutak, 1938, p.67), whereas, in cases of social capital, they may be weak (Granovetter, 1973). Finally, as all of its proponents use the concept, social capital inheres in individuals (Portes & Landolt, 1996), while communitas is a shared product of the collective.

Communitas differs from pre-existing capitals, then, in four ways. First, unlike capitals, it is not typically grounded in durable networks and institutional orders. Disasters disintegrate these everyday social structures, changing the environment and social dispositions. The “rules of the game” are reconfigured. Stable systems of rules and predictable behaviors no longer necessarily obtain. Communitas seeks to cope with this new normal, arising as a necessary response to wholesale change (Haney, 2018). Another way of saying this is that it is novel, spontaneous and emergent. Second, and as a corollary, we can say that communitas is more context- and purpose-specific than regular social capital. Third, we must pay attention to the precise contexts in which it emerges: it is a group formation that emerges under conditions of extreme stress. Fourth, capitals are usually deployed to secure individual or group advantage. For Bourdieu (1986), they are the primary mechanism through which privilege and social order are reproduced. Identities are ordinarily based on borders and exclusions, part of who you are is who you are not. Not everyone in a given region will be male, white, middle-class, heterosexual and able-bodied, for example. Disasters, by contrast, are all-inclusive. All those within the region will be afflicted (albeit to varying degrees). This binds survivors. Consequently, the strategic and self-interested investment/return motivations that define social capital (Colclough & Sitaraman, 2005, p.479) are absent here.

## Clarifying communitas II: Cognate situations

Bakhtin (1968), Solnit (2005) and Turner (1969, 1986) show that the utopian desire for a ludic “second life” beyond the world of exploitative hierarchical relations is a common human impulse. Analyzing this social logic will give us additional theoretical purchase on communitas. As they all note, societies create, or have forced upon them, “times out of time” where the normal rules cease to apply or fail to work. Turner identifies these liminal egalitarian moments in rites of passage and millenarian movements, Bakhtin in carnivals, while Solnit discovers that the same applies in disasters. Although disasters constitute a very different social situation, as with Turner’s observations of communitas and Bakhtin’s of carnival, during disasters the present is privileged over past and future, civil society over formal structures of state, the social over the individual, and the public over the private.

### Subjunctive moods

As already noted, we can detect utopian moments within disasters, discernible times when the population are in what Turner (1986) calls “the subjunctive mood”. He distinguishes subjunctive moods from “indicative” ones that relate to the mundane world of what is. In contrast, subjunctive moods gesture towards what *could be.*^10^ Turner said these were akin to disasters, in that they are breeching events where the ordinary operations of society are transgressed. The primary signal is crisis. There follow attempts to repair the social fabric. Groups may decide to reintegrate, or they may prefer to go their separate ways. Reintegration, even if sought, may prove unsuccessful under the radically changed circumstances, in which more revolutionary measures may come about. In these liminal moments, a space is opened up in which to think the social afresh and create new worlds. Wrote Turner (1986):

Just as the subjunctive mood of a verb is used to express supposition, desire, hypothesis, or possibility, rather than stating actual facts, so do liminality and the phenomena of liminality dissolve all factual and common-sense systems into their components and “play” with them in ways never found in nature or custom (p.25).

Turner was not explicitly writing about disaster, but his points have been repeatedly verified in disaster situations, leading to the paradoxical point that disasters can provide grounds for optimism (Oliver-Smith, 1999b).^11^

Turner (1969) points out that “for individuals and groups, social life is a type of dialectical process that involves successive experience of high and low, communitas and structure, homogeneity and differentiation, equality and inequality” (p.97). His discussion of communitas, which he describes as a condition of “anti-structure”, resonates with Bakhtin’s work on carnival, which also refers to the suspension of social hierarchy.^12^

### Carnivals

According to Bakhtin (1968), medieval and Renaissance life was dominated by a “two world” condition, one being the world of church and state, the other, a world beyond official authority, the time of carnival. During carnival, the different modes of being enforced by rigid class and status differentials disappeared. Society became homogenous. As Bakhtin makes plain, carnival was not watched, it was lived. Universal and inclusive, it was by the people, of the people and for the people: “carnival celebrated temporary liberation from the prevailing truth and from the established order; it marked the suspension of all hierarchical rank, privileges, norms, and prohibitions” (Bakhtin, 1968, p.10). Carnival was the masses’ “second life”, a utopian time of liberty, fraternity, equality and abundance. As with Turner’s subjunctive mood, it gestured to what was possible, what yet might be. Bakhtin (1968) described it as “the feast of becoming, change, and renewal” (p.10). And, as with disaster, carnival undoes the ruling orthodoxies, what Bakhtin (1968) called the “ready-made and completed… This carnival spirit offers the chance to have a new outlook on the world, to realize the relative nature of all that exists, and to enter a completely new order of things” (p.11, p.34).

### Disasters

Solnit (2005, p.36) brings Bahktin’s work directly to bear on disasters, making points that clearly connect with Turner as well. Her book-length study examined a range of historical and contemporary accidents and disasters: the 1906 San Francisco earthquake, the 1917 explosion of the ship *Mont Blanc* in Nova Scotia, the Mexico City earthquake of 1985, 9.11 and Hurricane Katrina. In each case, she identifies links between disaster, carnival and the desire for new and better forms of communal being. Disasters create the space for new types of priorities, bringing forth a state of being for one another. They “provide an extraordinary window into social desire and possibility, and what manifests there matters elsewhere, in ordinary times and in other extraordinary times” (Solnit, 2009, p.6).

For Solnit, disasters reveal who we are. Our responses to disasters show that we are resilient and generous, committed to the possibility of doing things differently, desiring of human connection and purpose (Solnit, 2009, pp.305–306). Disasters bind. Those in peril are rescued, the hungry are fed, the homeless are sheltered and the lonely are cared for. There is a kindness to strangers. Individuals band together for the collective good: “human beings reset themselves to something altruistic, communitarian, resourceful, and imaginative after a disaster” (Solnit, 2009, p.18; see also Casagrande et al., 2015, p.353).

## Some are more equal than others: Communitas, capitals and differential disaster response and recovery

Yet communitas is not an all-or-nothing affair. There can be varying levels of it *within* disasters. Some are accepted as community members or supported as deserving victims, while others struggle for recognition and resources. For these reasons, it makes sense to bring the discussion of communitas into productive dialogue with scholarship on social capital to explain differential outcomes post-disaster. This will help to identify barriers to the development of communitas.

While communitas speaks to notions of people power and equality, it is clear that some people might be more powerful – and hence more “equal” – than others. This is important as material inequality is one of the biggest drivers of vulnerability (Bolin, 2007; Tierney, 2006). Indeed, disasters do not permanently eradicate the obdurate features of social structure. Buildings crumble, but vested interests, institutions and connections can endure. The systems that disburse prejudice and privilege are remarkably resilient (Tierney, 2014, p.237). Victimology records this: the isolated, weak, minorities and the less wealthy consistently fare worse in disaster situations (Matthewman, 2015, pp.20–21; see also Phibbs et al., 2018).

Disaster and resilience researchers frequently draw upon social capital theory to explain this. Its role in bringing people together to facilitate effective disaster response and meaningful recovery is frequently highlighted (Tierney, 2014). Social capital has many benefits, including the ability to access resources and work collectively towards recovery. Conversely, those with fewer social connections are far more likely to end up as disaster victims and to struggle with recovery (Dynes, 2005). Aldrich’s (2012, pp.31–34) scholarship is exemplary in this regard. He conceptualizes social capital in three different forms: bonding (within networks or homogeneous communities, i.e., people who are close), bridging (between networks or more loosely connected communities) and linking (across vertical gradients, i.e., connecting people with official authority figures).

Limited literature is available on the conditions that increase and decrease inter-community and macro-level bridging or linking capacities, although there is work suggesting that socioeconomic status (SES) and race are significant predictors of social capital and the distribution of resources in recovery. Hawkins and Maurer’s (2010, p.1785) study of Hurricane Katrina argues that SES and race remained significant barriers to bridging and linking social capacities. In general, the more socially advantaged have broader connections (better bridging and linking capacities as more localized bonding social capital is often less important), while the more socially vulnerable have stronger localized social connections and better bonding social capital capacity (see Rivera & Nickels, 2014, p.185).

Here it makes sense to draw on the work of Bourdieu (1986). His scholarship illuminates the network effects that accrue from the combined forms of capitals that create social advantage. Bourdieu (1986) uses capital theory to refer to almost any resource: capital might be economic (e.g., cash and financial assets), cultural (e.g., attitudes, education, knowledge, language, past experience, skills), social (e.g., networks and group memberships), or symbolic (e.g., visibility, stereotypes, social status). These capitals can intersect with each other and convert into/from the other(s) (Bourdieu, 1993). For Bourdieu (1986, 1989), capital possession determines social position, particularly within the related domains of education and labor market. It is equally applicable to disaster zones. The total amount, quality and interplay of capitals combines to determine resource-level and social standing. This impacts upon individuals and communities’ disaster experiences. Those with more capital possession will likely experience higher resilience and vice versa (Obrist et al., 2010; Wilson, 2012). Importantly, for Bourdieu (1993), the value of capital is determined through the interplay between field and habitus. Fields are the specific social spaces where individuals compete for the distribution of capital. Habitus refers to the internalized, taken-for-granted elements of social structure. These *structural* factors are typically disregarded in accounts of social capital.

Individuals are seen as strategic and rational social agents who acquire particular types of capital that are valuable in, and applicable to, particular social situations (fields). In a field, certain capitals are valuable, but, in different fields (or rapidly changing fields during disasters), the same capital may be worthless. In other words, agents do not simply act in the world. They are also constrained by habitus and by context (field).

In addition to alerting us to the resilient system of social structure, Bourdieu’s theory offers another important element: the role of symbolic capital (defined as standing, value, recognition, prestige). External support may focus on those most visible or deemed most deserving. Stereotypes, misrepresented or manipulated via media, may become a strong predictor of who will accrue capital by gaining financial aid and other forms of assistance (Lee et al., 2015; Scanlon, 2007). Some communities with better symbolic capital such as the Vietnamese Catholic community in Eastern New Orleans after Hurricane Katrina (Leong et al., 2007) and the Filipina community in Tohoku (Shunhee Lee, 2012) following the 2011 Great East Japan Earthquake and Tsunami garnered more, and more positive, public attention than other minority groups, resulting in more external support and extended social and political networks.

Unsurprisingly, stigmatized communities struggle to gain help (Dyson, 2006; Leong et al., 2007). The fortunes of the Vietnamese Catholic and Filipina communities in the cases at hand compare favorably to communities with lower levels of symbolic capital such as African-American communities. More than a million, predominantly black, people were displaced from New Orleans. To call them second-class citizens does not do justice to their media treatment. In quick order, mainstream news outlets took to calling them refugees. No longer fellow Americans, they were stateless people. Gralen B. Banks, a community activist, rhetorically enquired in Spike Lee’s (2006) epic documentary: “Damn, when the storm came in it blew out our citizenship too?”

## Communitas denied

Communitas is often observed in disasters, but it is not *always* observed (Richardson et al., 2014, p.211). There is no iron law guaranteeing its emergence. Communitas encounters limits. Identifying them has important implications for effective DRR strategy. Richardson et al.’s (2014) study of a small Texan community struck by Hurricane Ike, for example, draws our attention to “community characteristics, such as leadership, demographics, length of community residence, community size, and sense of community or closeness during everyday circumstances” as determining characteristics for “foster[ing] communitas during disasters” (p.211). It could also be the case that the scale of a disaster completely overwhelms a community’s coping abilities (Barton, 1969). Under such conditions, communitas is far less likely to emerge.

Given its potential to be a powerful resource for recovery and a means to build back better, it is important to identify conditions that constrain it. We draw upon Klinenberg’s “social autopsy” of the 1995 Chicago heatwave and the scholarship on toxic spills and related “natural hazard triggering technological” (natech) disasters to do so. Several explanatory factors for communitas’ failure to eventuate loom large: family structures, community dynamics, levels of civic support, the severe degradation of social infrastructure and the “corrosive community” that can ensue from toxic spill events, principally through the nebulous nature of these threats. In such cases, detecting and ameliorating harm can be difficult, as can apportionment of blame and securement of proper recompense.

### No joy in disaster I: Depleted social infrastructure

Severe deprivation means that some communities cannot self-organize as, to all intents and purposes, there is no community to be organized. People exist under conditions of co-presence rather than being enmeshed in supportive social relations. Klinenberg’s (2002) study of the 1995 Chicago heatwave is instructional here. It brings social ecology into consideration by comparing two climatically, demographically and socioeconomically similar communities: North and South Lawndale. Despite their affinities, residents in these two locations experienced markedly different outcomes. North Lawndale had ten times the rate of fatalities of its neighbor. North Lawndale’s population was 96% African American, South Lawndale’s population 85% Latino. The hardest hit areas of Chicago were poor, black and crime riddled. In contrast, Latino communities had the highest survival rates. To explain this disparity, we need to examine social infrastructure.

North Lawndale began losing its industrial base in the 1950s. Other commercial activities and public amenities soon followed. The underground economy usurped the formal one. Large swathes of the population left the area. As they dispersed, support networks stretched, sometimes to breaking point. More transient (and reclusive) people populated the hitherto stable neighborhoods. City authorities also withdrew. The conditions that allow drug dealers to feel safe – open corners, empty lots, high grass, bad lighting – make others feel unsafe. When the heatwave came, poverty stopped people from cooling off inside. They either did not have or could not afford to use air-conditioning. Civic neglect and fear of crime compounded the problem. There was nothing to go out to, and open spaces were viewed as threatening.

The neighborhood to the immediate south was an entirely different world. South Lawndale escaped the ghettoization that took place in North Lawndale, the vicious cycle of neglect, civic withdrawal, decline, vandalism and violence leading to atomization. Moreover, it was boosted by constant Mexican and Central American in-migration. During the period in which North Lawndale’s population halved, South Lawndale’s grew by 30%. The high population density gave the place vibrant street life and plentiful commercial activity. This fostered sociability and mutual care. In South Lawndale, it was far harder to fall through the cracks. People felt safe in public. When the heatwave came, the elderly comfortably ventured outside. In doing so, others could keep a neighborly eye on them and they had places to go. They could cool off in air-conditioned shops (Klinenberg, 2002, pp.79–128).

### No joy in disasters II: Corrosive communities

The above discussion shows that depleted social infrastructure corrodes community spirit and hence communitas. The same can be said of the “new species of trouble” that comes from modern toxins. Kai T. Erikson (1995, p.141) argues that poisons create their own peculiar fears. He outlines three reasons for this. First, there is the undefined nature of their harm. They present new challenges regarding detectability and duration. Toxins do obvious physical damage. They have profound psychological impacts too. Fear is intensified as these threats typically evade bodily protection mechanisms: our senses. We do not know when we are at risk. It can be years before there is decisive confirmation of contamination. Moreover, they seem to be disasters without conclusion. Toxic disasters have uncertain lifecycles. They do not simply begin, exist and then end (Erikson, 1995, p.147).

Second, toxic events can create “corrosive communities” in which social bonds are weakened by prolonged litigation, victim blaming and community division, all of which undermines the prospects for collective action aimed at recovery (Freudenburg, 1997). These technological disasters are often highly complex. Identifying causal chains may not be easy; consequently, legal settlement may be difficult. This brings its own trauma.

Third, these disasters display widespread levels of recreancy. Just as health fails, so too do institutions. Numerous officials, agencies and institutions can let people down, compounding social stress (Picou et al., 2004). Ill-defined, imperceptible and therefore difficult to counter, communities tend to display profound feelings of inadequacy in the face of toxic events, at being out of control and being abandoned by authorities.

Matters can be further complicated in the case of natech disasters, which combine natural hazards with socio-technical threats, as in the 2011 Great East Japan Earthquake. This triggered a tsunami, which inundated a nuclear power plant, leading to a meltdown. Knock-on effects included damage to the national electricity grid and disruption to worldwide supply chains. Natech disasters can often produce: “(1) a contested discourse regarding who was responsible; (2) chronic social disruption, anger, mental health problems, and the loss of resources for victims; (3) delayed restoration and recovery for impacted communities; (4) ambiguity of harm; (5) sociocultural disruption; and (6) litigation stress” (Picou et al., 2007, p.5).

## Conclusion

This article has theorized communitas following disasters. We have observed the reasons for its emergence (foregrounding the shared nature of disaster and the frequent insufficiency of official resources), factors that mediate disaster recovery (via the role of Bourdieusian capitals), and those that outright prevent it (depleted social infrastructure and ongoing toxic events). In so doing, we have noted differences between communitas and similar terms. Communitas differs from the community of fate in that social relations are horizontal, bonds are empathic, altruistic acts are more pronounced, and – like carnival – a palpable sense of joy is often discernible. Communitas also differs from social capital in that it is short-lived, context-specific, experientially based, inclusive and transformational.

Given the potential for communitas to be a powerful resource for DRR and a means to build back better, we should look to enhance it. Indeed, community groups often succeed where governments fail. For example, Occupy Sandy, a collective that formed to provide relief in the aftermath of Hurricane Sandy, was roundly acknowledged to have provided relief more effectively than either federal agencies or formal non-governmental organizations (Burke & Yeager, 2012; Dawson, 2017).^13^

We are still a long way from being able to create communitas at any time, in any place, or at any scale. Moreover, communitas only ever appears to be temporary (Casagrande et al., 2015, p.353; Hearn, 1980, p.316). Yet Atsumi’s (2014, p.152) study shows “that the state of post-disaster ‘paradise’ can be maintained, at least intermittently” by donating funds and volunteering labor to help those struck by other disasters. This creates social bonds between groups even when the direct experience of a particular disaster is not shared. Similarly, it has been observed that media exposure can do much the same (O'Brien & Mileti, 1992). This suggests that fundraising, volunteering and responsible media representations of disasters are all helpful. Even potentially corrosive communities have demonstrated that they can collectively cope with toxic events if they are sufficiently motivated, skilled and organized (Gunter et al., 1999; Kaniasty & Norris, 2004). Clearly institutional systems that support community organization are to be welcomed and those that encourage robust regulatory oversight, with strong “polluter pays” policies and swift resolution of legal claims would be beneficial too.

By developing social infrastructure and social capital, we build resilience and we create a potent force for disaster recovery (Klinenberg, 2018). Carlton and Vallance (2018) have shown that in post-disaster Christchurch, for example, transitional urbanism projects undertaken by the community function as important sites for social capital generation (and see Aldrich & Meyer, 2015, pp.262–263 for further suggestions).

While there is merit in all of the abovementioned suggestions, if we truly want to stay in the subjunctive mood (Turner), keep the carnival going (Bahktin) and extend extraordinary community (Solnit), we need to permanently eradicate hierarchy. This requires a properly structural response. The only way to ensure an enduring society of equals is through the redistribution of resources. This will need to apply to all four forms of capital (Bourdieu).

## Footnotes

1 The United Nations International Strategy for Disaster Reduction (UNISDR) (2009) defines vulnerability as: “The characteristics and circumstances of a community, system or asset that make it susceptible to the damaging effects of a hazard”. It defines resilience as: “The ability of a system, community or society exposed to hazards to resist, absorb, accommodate to and recover from the effects of a hazard in a timely and efficient manner, including through the preservation and restoration of its essential basic structures and functions”.

2 It is telling that the only book-length study of communitas to synthesize a range of case studies, theorize its emergence and utility, and make generalizable claims about it, was produced by an outsider to the field: Rebecca Solnit’s (2009) *A paradise built in hell: The extraordinary communities that arise in disasters*.

3 Despite tying notions of communitas to visions of the catastrophic, Victor Turner (1969, p.154) never explicitly connected his concept to disasters. His wife, Edith Turner (2012, pp.73–78, p.83) did, recounting Linda Jencson’s work on the Dakota floods of 1997, and Carl Lindhaus’ work on Hurricane Katrina. She saw clear advantages for disaster response and recovery from communitas and, unlike her husband, did not believe that communitas was necessarily only temporary, writing: “The benefits of communitas are quick understanding, easy mutual help, and long-term ties with others” (E. Turner, 2012, p.3).

4 The largest independent global review of disaster risk undertaken at the local level found that those at risk of disasters are continually ignored when it comes to making decisions about disaster prevention in their communities. Only a meagre 16% of respondents said that they felt included in risk assessment exercises, mitigation plans or actions to minimise hazards (Global Network of Civil Society Organisations for Disaster Reduction (GNDR), 2019).

5 Given social theory’s historic tendency to focus on stability and order (Guggenheim, 2014, p.4; Turner, 1994, pp. 172–174), mainstream theorists may also find theorizing breakdowns and ruptures of interest.

6 As George Monbiot (2020) chronicles, we are seeing such acts constantly, from the young volunteers in Hyderabad who are provisioning the city’s precarious workers with food packages, to the helpers in Wuhan who are ferrying essential medical workers between hospital and home, to the programmers in Latvia who organized a hackathon to create optimal face shield components for 3D printers. Monbiot (2020) states: “[t]he shift is even more interesting than it first appears. Power has migrated not just from private money to the state, but from both market and state to another place altogether: the commons. All over the world, communities have mobilized where governments have failed” (para.2).

7 The word “altruism” is a sociological neologism, coined by Auguste Comte. Comte felt compelled to counter assertions that human nature is essentially selfish. He believed that people routinely suppress egoistical desires in service of the collective, to a degree unseen in any other species. He also felt that the work we do is largely undertaken for others. He coined the term “altruism” to describe this, which is derived from the Italian “altrui”, meaning “of others”, “to others”. For Comte, altruism was composed of three core instincts: attachment, benevolence and veneration. In a fit of modesty, he placed his revelation alongside Copernicus’ work on the Earth’s motion, declaring it “the chief discovery of modern science” (Quoted in Dixon, 2008).

8 Some disaster researchers have dubbed these collective responses “altruistic community” (Barton, 1969; Kaniasty & Norris, 2004).

9 For more on the affinities of collective effervescence and communitas, see Olaveson (2001).

10 COVID-19 provides us with a case in point. Most Britons surveyed in a YouGov poll hoped that both they and their country would change for the better following the pandemic. Only 9 % of those questioned wanted to return to the status quo ante (Binding, 2020). Similarly, a global IPSOS survey of 21,000 adults in 27 countries on behalf of the World Economic Forum showed that almost 90% of respondents wanted a fairer world post-COVID-19. Of those questioned, 86% wanted to see significant change, particularly in terms of social and environmental justice (IPSOS, 2020). As Derek Thompson (2020) wrote, “Calamity can stir us from the trance of complacency and force us to ask first-principle questions about the world: What is a community for? How is it put together? What are its basic needs? How should we provide them?”

11 Oliver-Smith (1999a, b) points are informed by his decade of research on the Peruvian earthquake of 31 May 1970, which badly affected the Yungay region. An avalanche obliterated the city. Almost immediately, survivors rendered first aid to the injured and searched for the missing. Isolated for days, they formed groups to source provisions, construct shelters and care for the wounded. Those with spare food or livestock donated it to the collective. Previous distinctions like urban or rural, Indian or non-Indian, rich or poor, were eradicated. Once outside aid began to enter, the collective solid bonds began to loosen. Even so, the community remained sufficiently organized to resist central government plans to relocate their city. They successfully rebuffed the politicians in the metropolis and rebuilt their provincial capital across a two-year period. The hardship was real, but so too was their shared sense of purpose. “Perhaps, there needs to be less consideration toward the delivery of outside aid”, Oliver-Smith, (1999a, b, p.168) concluded, “and more attention devoted to devising culturally appropriate ways to nurture the potentials represented by post-disaster solidarity”.

12 Of course, there are also key differences between disaster and carnival. The former is a response to the catastrophic, the unexpected, the novel and the emergent. Actors must improvise with imperfect information and adjust to constantly changing demands and conditions. By contrast, carnival is scheduled, large amounts of coordination take place prior to the event, and actors already share understandings of appropriate roles and behaviors through long-established histories of practice.

13 See also: Matthewman (2015, p.166), Preston and Firth (2020), Roasa (2013), and Monbiot’s quote in footnote for further examples.
